# Lemming and Vole Cycles: A New Intrinsic Model

**DOI:** 10.1002/ece3.70440

**Published:** 2024-10-21

**Authors:** Elizabeth A. Levay, Helen Nasser, Matthew D. Zelko, Jim Penman, Terrance G. Johns

**Affiliations:** ^1^ School of Psychology and Public Health La Trobe University Melbourne Victoria Australia; ^2^ Epigenes Australia Pty Ltd Melbourne Victoria Australia

**Keywords:** delayed‐density dependence, epigenetics, hormones, lemming, population cycles, vole

## Abstract

It is 100 years since the first paper described the multiannual cycles in Arctic rodents and lagomorphs. The mechanisms driving population cycles in animals like lemmings and voles are complex, often attributed to extrinsic factors, such as food availability and quality, pathogens, parasites and/or predators. While extrinsic factors provide insights into population cycles, none fully explain the phenomenon. We propose an underlying innate, intrinsic mechanism, based on epigenetic regulation, that drives population cycles under harsh arctic conditions. We propose that epigenetically driven phenotypic changes associated with sexual development, growth and behaviour accumulate over time in offspring, eventually producing a phase change from rising population density to eventual population collapse. Under this hypothesis, and unlike previous hypotheses, extrinsic factors modify population cycles but would not be primary drivers. The interaction between our intrinsic cycle and extrinsic factors explains established phenomena like delayed‐density dependence, whereby population growth is controlled by time‐dependent negative feedback. We advocate integrating a century of field research with the latest epigenetic analysis to better understand the drivers of population cycles.

## Graphical abstract

It is 100 years since the first paper described the recurring nature of Arctic population cycles, yet the mechanistic drivers of these are still not well understood. This paper challenges researchers to consider a new paradigm, with reference to the role of epigenetics in the cycling process. We provide a testable hypothesis describing a series of hormonal/epigenetic steps that form an intrinsic cycle.

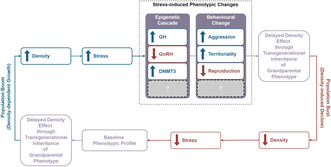

## 1 Introduction

Intrinsic physiological rhythms lead to relatively short cycles driven by the tide (circatidal) and the earth's 24‐h rotation (circadian). Longer cycles are mediated by the lunar cycle (circalunar) and seasonal variation (circannual). Extensive research has elucidated many of the intrinsic genetic, epigenetic and hormonal mechanisms driving these intrinsic cycles/rhythms (Fagiani et al. 2022; Hafker et al. 2023; Lincoln 2019; Neumann and Kaiser 2023). While the physiological mechanisms of these varied cycles are intrinsic, changes in environment and behaviour can influence their timing and amplitude (Andreatta and Tessmar‐Raible 2020; Fagiani et al. 2022).

Despite our understanding of biological rhythms, certain species exhibit cycling enigmas that still lack a satisfactory explanation. For instance, how do periodic cicadas maintain their 13‐ or 17‐year cycles spent underground as nymphs before emerging simultaneously (Sota 2022)? Similarly, at a population level, lemmings, and their near relatives the voles (both belonging to the subfamily Arvicolinae), undergo a significant behavioural shift as part of their regular 3‐ to 5‐year population cycles. They transform from being wary and tentative creatures to becoming impetuous and exploratory ones (Andreassen et al. 2013; Gaines and McClenaghan Jr. 1980; Krebs 1992), behavioural changes that are associated with dramatic changes in population density (Ims, Yoccoz, and Killengreen 2011; Oli 2019). While the harsh arctic and sub‐arctic conditions are strongly associated with these multiannual cycles, the exact extrinsic factors driving this regular change in behaviour remain unknown. Even less is known about the intrinsic physiological and epigenetic changes associated with these multiannual cycles.

In this opinion piece, we briefly review the current state of literature with respect to our understanding of multiannual cycles, with a particular emphasis on lemmings and voles. We highlight the limitations of previous field‐based methodologies, and the dearth of studies directed at understanding the intrinsic mechanisms underpinning these cycles. Based on our existing knowledge base, we propose a new, testable hypothesis for multiannual cycles focusing on stress‐induced epigenetic drivers, and concomitant phenotypes, that are inherited transgenerationally. Field research should remain an indispensable part of understanding the mechanisms that drive population cycles. However, it is time to augment these studies with detailed epigenetic and hormonal studies to better understand the dramatic changes in biology and behaviour across the cycles.

### 1.1 Overview of Multiannual Population Cycles

**FIGURE 1:**
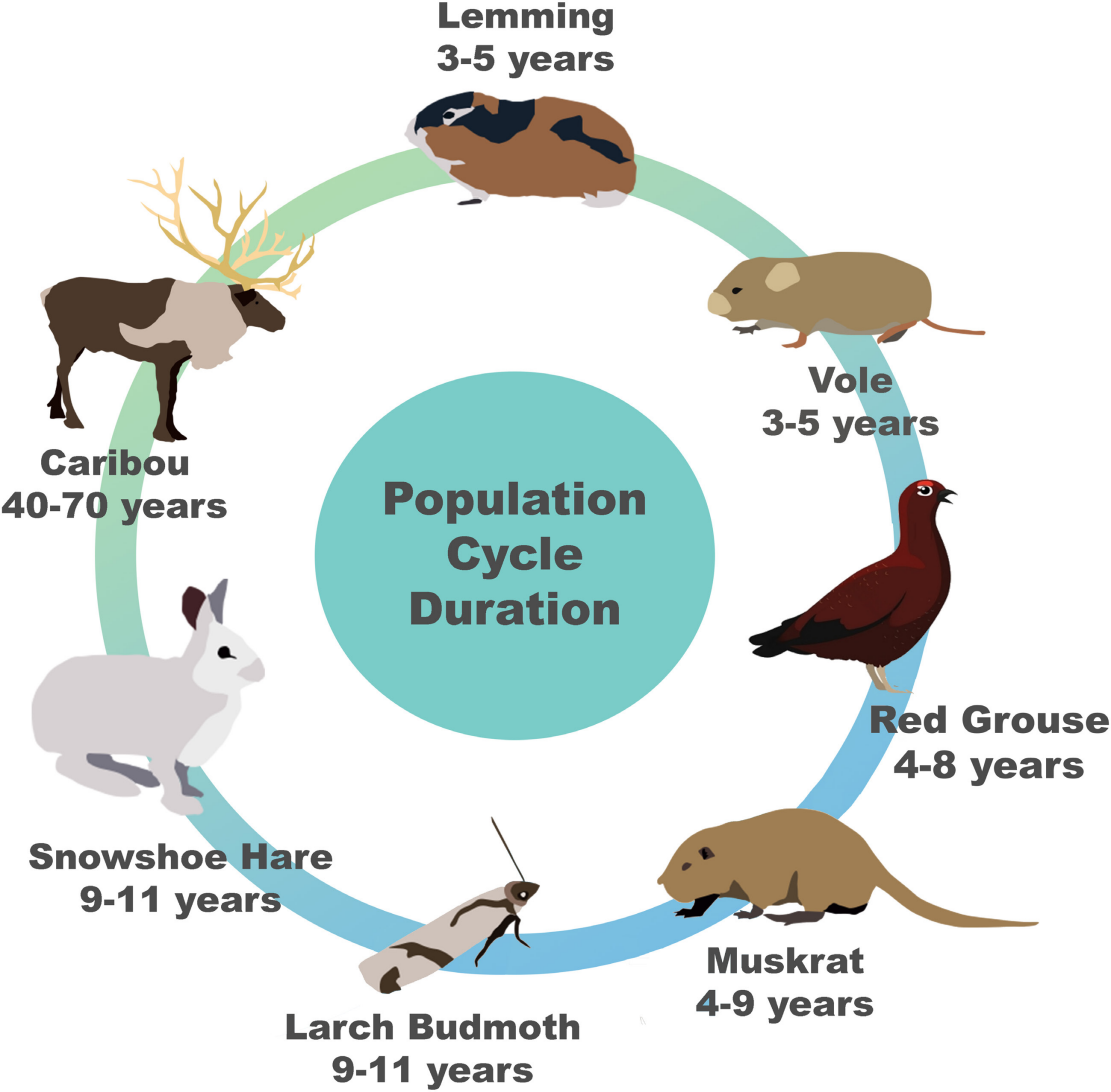
Population cycle duration for selected species.

Multiannual cycles are characterised by periodic fluctuations in abundance, exhibiting distinct cyclic phases—increase, peak, crash and low phase (Krebs and Myers 1974). Such cycles occur with striking regularity and are present in multiple taxa, from the larch budmoth to lemmings and voles, muskrats, snowshoe hares, red grouse, and some larger mammals such as caribou, with cycle lengths ranging from 3–5 years (lemmings and voles; Andreassen et al. 2021), 4–8 years (red grouse; Moss and Watson 1991), 4–9 years (muskrats; Erb, Stenseth, and Boyce 2000), 9–11 years (larch budmoth and snowshoe hare; Baltensweiler 1993; Krebs et al. 1986) to 40–70 years (caribou; Gunn 2003) (Figure 1). It is important to note that multiannual cycles do not always occur in some species. For example, a review of population cycles in North American red‐backed voles (Boonstra and Krebs 2012) identified seven studies of which two showed a population cycle of 3–5 years; cycles were not identified in the other studies. This overview highlights the challenges of identifying consistent results across the thousands of population cycle publications. Therefore, we have tried to highlight the aspects of population cycles where there is broad agreement (see Krebs 2013; Oli 2019 for reviews).

As well as regular periodic oscillations, multiannual cycles are characterised by highly variable amplitudes. In Northern Hemisphere Arvicoline rodents, such as lemmings and voles, the amplitude of their cycles can be dramatic. Density increases of 2–3 orders of magnitude from the low phase are observed, with peak densities reaching up to 1000 individuals/ha (Andreassen et al. 2021). Additionally, the amplitude of the cycle is affected by the severity of seasonality, with harsher and more protracted winters associated with more intense amplitudes (Andreassen et al. 2020; Saitoh, Stenseth, and Bjørnstad 1998; Stenseth et al. 2003), though see Korpela et al. (2013) where pronounced vole cyclicity in Finland was apparent in climates with long and cold winters but also in climates with relatively long and warm summers.

**FIGURE 2:**
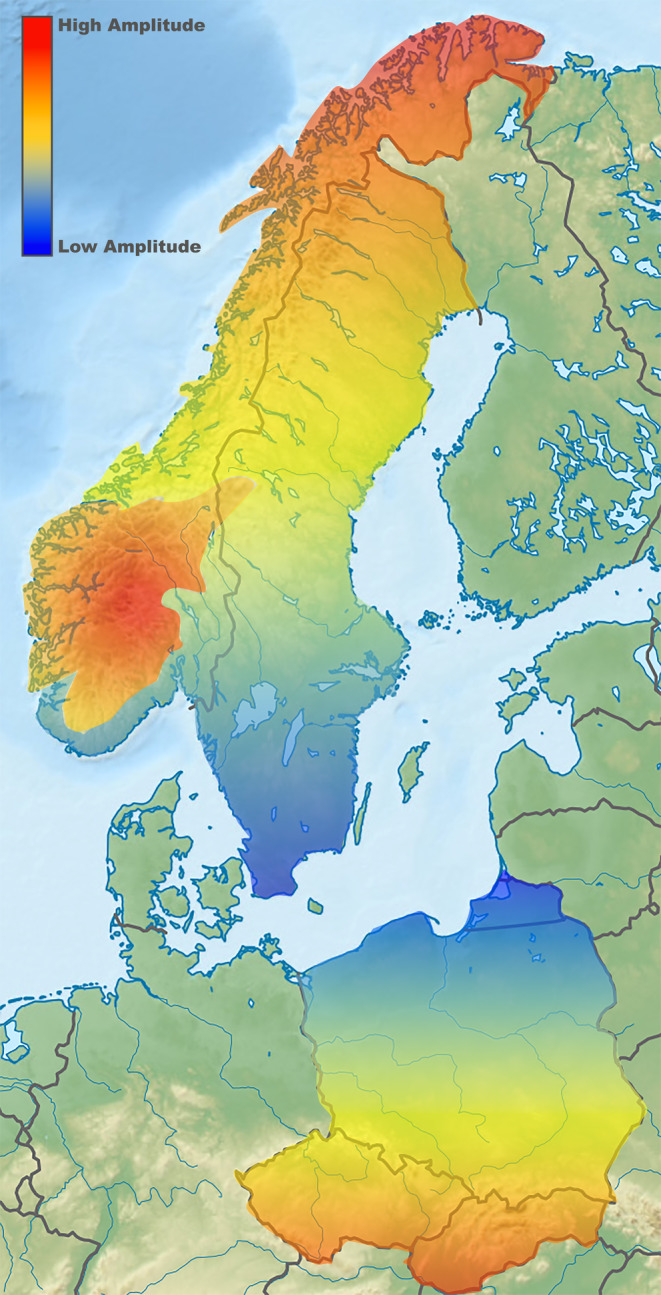
Northern and Southern European population cycle amplitude gradients (*Myodes* and *Microtus* voles). North–south, altitudinal and coast‐inland gradients are reflected. *based on (Andreassen et al. 2021; Hanski and Henttonen 2002; Ims, Yoccoz, and Killengreen 2011; Tkadlec and Stenseth 2001; Turchin and Hanski 1997).

The seasonal impact on cycle amplitude typically reflects a latitudinal effect with cycles decaying at lower latitudes (Saitoh, Stenseth, and Bjørnstad 1998), though coast‐inland (Tkadlec and Stenseth 2001) and altitudinal gradients (Ims, Yoccoz, and Killengreen 2011) have also been documented (Figure 2). The latter observations also likely reflect variable seasonality, with harsher and longer winters inland, as compared to the coast, and at higher altitudes. In addition to these spatial discrepancies, amplitude dampening and, in some instances, cycle degradation has been observed within cyclic populations over time within the same geographical locale, a corollary linked to climatic warming (Bierman et al. 2006; Hörnfeldt, Hipkiss, and Eklund 2005; Kausrud et al. 2008).

Differences in the rate of change, magnitude, and periodicity of cycles are also noted between species. For instance, the Norwegian lemming exhibits a steeper increase phase (Ims, Yoccoz, and Killengreen 2011) and sharper peaks (Turchin et al. 2000) than voles in Northern Fennoscandia. Conversely, there are similarities in cyclic dynamics between species, such as regional synchrony of species occupying overlapping geographical areas, and wide geographical synchrony of cycles across vast areas (Korpimäki and Krebs 1996). For example, synchronicity over thousands of square kilometres has been observed in two lemming species in eastern Canada (Krebs 1963). Such synchrony suggests a common underlying cause or causes. Whether this be large‐scale climatic factors across regions, trophic interactions or some inherent mechanism remains to be determined.

An additional characteristic of cyclic populations are differences between the cycle phases in the demography and behavioural phenotype of the rodents themselves, a collection of attributes coined the ‘biological definition’ of cyclic fluctuations by Krebs (1996). Specifically, phase‐specific differences in the size of animals, age at sexual maturation, reproductive rates, sex ratio, survival, sociality and propensity for dispersal have all been documented (Boonstra 1985; Boonstra and Krebs 1979; Fauteux and Gauthier 2022; Johnsen, Devineau, and Andreassen 2019; Novikov, Panov, and Moshkin 2012). For instance, animals in the peak‐density phase of the cycle are 20%–30% larger than animals in the low‐density phase of the cycle (Boonstra and Krebs 1979; Oli 1999). Moreover, animals typically display lower reproductivity (Novikov, Panov, and Moshkin 2012; Oli 1999) and increased aggression (Krebs 1970) at peak density.

An important concept within cyclic population research is density‐dependent growth. It describes how population size varies in response to changes in population density. Population growth can be affected by direct density‐dependent factors. For instance, as population density rises, mortality rates often increase due to factors such as limited food supply, higher likelihood of disease transmission, and intensified competition for mates. These negative feedback mechanisms can decelerate population growth or, in some cases, lead to a population decline. Cyclic populations are also conditional on delayed‐density dependent mechanisms, whereby population growth is influenced by factors that operate with a time lag (Oli 2019). For example, it may take time for food supply to be depleted or for predator numbers to respond to changes in prey abundance. This concept of delayed‐density dependence has been used to explain small rodent population cycles. The causes, thus far, are suggested to include trophic interactions, namely, vegetation‐rodent, rodent‐parasite or rodent‐predator interactions (Oli 2019). In each of these interactions, there is potential for current population size to be influenced by past population size.

### 1.2 What we Know (and don't Know) About the Mechanistic Drivers of Lemming and Vole Cycles

The drivers of multiannual cycles in small mammals have been the subject of intense research. Charles Elton (Elton 1924) pioneered the investigation in his seminal essay outlining his theory regarding the cause of periodic fluctuations. His insights are considered the cornerstone of contemporary ecology (Lindström et al. 2001) and inaugurated over 100 years of intense research. Despite the plethora of data and countless hypotheses generated, the cause of these cycles is still largely unresolved. We will not go into detail here about these hypotheses and associated evidence as this has been expertly reviewed (Andreassen et al. 2021; Kelt et al. 2019; Krebs 1996, 2013; Oli 2019). In brief, proposed and tested mechanistic drivers of cycles include predation, food availability, disease, social processes, stress and physical factors. These factors can be categorised into extrinsic factors, such as climate, predators and food availability, and intrinsic factors that are inherent to the population, including physiology, stress and behaviour.

The majority of research interest has focused on extrinsic modulators of multiannual cycles, with the predation hypothesis being the most intensively studied (Kelt et al. 2019). This theory predicts that multiannual population cycles are caused by interactions between specialist predators and their prey. Experimental manipulation, such as predator removal and observational field studies have provided evidence and indications both supporting (Korpimaki et al. 2002) and rejecting this hypothesis (Graham and Lambin 2002). Similarly, field and observational studies and mathematical modelling manipulating food availability and rodent pathogens are equally inconclusive (see Oli 2019).

There are significant practical challenges with large‐scale field experiments. For instance, predator exclosures are extremely laboursome to build and maintain. In addition, small mustelids, like the least weasel, can also move in and out of the exclosures. Furthermore, simple modelling and field experiments cannot possibly account for the intricate complexity underlying multiannual population cycles. Advanced population models that utilise stochastic, delay or partial differential formulations may help to address temporal and spatial feedback in small rodent cycles (Anashkina et al. 2016; Otto, Fagan, and Li 2022). Critically, such models suffer a similar issue as data‐driven analysis, namely a paucity of reliable, timely, and rich data sources from field experiments and observations. Therefore, increasing the frequency, breadth and accuracy of observational and experimental sampling in the field would aid in creating and validating more complex models.

To date, no experiment has unequivocally stopped a multiannual population cycle. While the capacity to conduct field experiments on the spatial and temporal scales required for eliminating cycle‐generating mechanisms is limited, existing data demonstrates that excluding environmental factors, such as removing predators and disease and ensuring plentiful food, does not eliminate cycles. Data suggests that environmental factors limit population growth but do not cause population cycles (Andreassen et al. 2021). This limitation suggests that the cause of cycles is within the animals themselves. This sentiment harks back to Elton's (1924) original supposition that the causes of cycles “lie either with the lemmings themselves or with their environment.” While he dismissed intrinsic modulators as untenable due to widespread synchrony across the Arctic, research on multiannual population cycles has returned to where it started.

The involvement of intrinsic mechanisms, or population self‐regulation, has received significantly less theoretical and particularly experimental attention than extrinsic modulators. Despite this, hypotheses that focus on intrinsic mechanisms have been proposed over the past 70 years. The central premise adopted by those propounding intrinsic mechanisms is that indefinite population increase is limited by a change in something inherent to the animals (Krebs 1978). These changes are either physiological or behavioural, and several specific regulatory mechanisms have been proposed. These include, but are not limited to, the stress hypothesis, the polymorphic behaviour‐genetic, or Chitty Hypothesis (largely discredited—Boonstra and Boag 1987), the maternal effects hypothesis, and the sociobiological hypothesis (see Kelt et al. 2019; Krebs 1978).

Given the difficulty in manipulating intrinsic factors, there is no conclusive evidence that these cause lemming and vole population cycles (Kelt et al. 2019; Oli 2019). Phase‐specific demographic and behavioural differences in phenotype, such as phase‐specific differences in the size of animals, age at sexual maturation, reproductive rates, sex ratio, survival, sociality and dispersal, certainly support intrinsic population regulation. However, the causes of these differences and their attendant underlying biological mechanisms are not well understood. Similarly, how these phase‐dependent factors affect the population cycles of small mammals remains equally enigmatic. Any hypothesis attempting to expound population cycles should account for these phase‐specific variations (Kelt et al. 2019; Oli 2019).

Potential biological candidates explaining phase‐specific phenotypic variations include epigenetic mechanisms. These would not have eluded Dennis Chitty had he lived and worked a few decades after his time. Epigenetic mechanisms include environmentally induced changes to gene expression, allowing rapid, heritable phenotypic alterations without altering the genome itself—the premise of Chitty's hypothesis. Specifically, epigenetic changes such as DNA methylation, the most characterised epigenetic marker, involve adding a methyl group to the DNA molecule. Importantly, while this modification does not alter the DNA sequence, it does impact how the DNA functions and, thereby, how genes are expressed. Furthermore, DNA methylation is a heritable trait (Nabais et al. 2023). Identifying these epigenetic modifications could explain the phase‐related differences in behaviour and physiology (Edwards et al. 2021). Additionally, we propose that the severe arctic conditions have led to the gradual synchrony of population phases by hardwiring this program of epigenetic changes into multiple species, thus becoming the underlying cause of population cycles.

Some recent research efforts have been directed toward determining potential epigenetic mechanisms underlying multiannual population cycles, though there is a relative paucity of studies. Evidence for epigenetic involvement has arisen from studies investigating the effect of the early life of female voles on the phenotypic outcomes (growth, reproductive characteristics and survival) of the subsequent generation (Sundell, Ylonen, and Haapakoski 2019; van Cann et al. 2019). Such studies demonstrate a direct influence of the early life of female voles on the phenotype and fitness of subsequent generations, suggesting an intrinsic proclivity to respond to environmental cues. Additional studies demonstrating altered gene expression of arginine vasopressin and oxytocin in the brains of voles at high density (Huang et al. 2021) and glucocorticoid receptors in the brains of female juvenile voles born at high density (Edwards, Palme, and Boonstra, 2023) also implicate underlying epigenetic mechanisms.

To our knowledge, only one study has attempted to investigate potential epigenetic mechanisms driving population cycles. In an enclosed vole population, juvenile female voles born at high population displayed reduced expression of gonadotropin‐releasing hormone (GnRH) in the hypothalamus as well as increased methylation of GnRH at two CpG sites (Edwards et al. 2021). These results support the hypothesis that the natal environment can drive selection for a low‐reproductive phenotype, underscoring an intrinsic driver of population cycles. In a commentary article on Edwards et al. (2021), Montgomery (2021) outlined a model whereby methylation of genes related to reproduction and social behaviour increases as the population peaks and decreases as the population crashes. He also proposes that the different levels of stress at the high and low phases of the population cycle help regulate this process. How this model might directly drive population cycles needs further elucidation.

## 2 A Proposed Intrinsic Cycle: Epigenetic Regulation of Lemming and Vole Multiannual Cycles

We propose that population cycles are driven by intrinsic epigenetic regulation in many species and that the harsh arctic conditions determine the periodicity and amplitude of the cycles. The presence of this functional intrinsic cycle drives alternating periods of timidity and population collapse, which starve predators, with periods of rapid growth and boldness, which allows them to colonise new areas (Andreassen et al. 2013; Gaines and McClenaghan Jr. 1980; Krebs 1992) before predators have had a chance to recover. Cycles become longer and more intense in environments that inflict periodic food deprivation while at other times providing ample resources for rapid population growth. Such conditions are characteristic of more northerly areas. When conditions are milder and resources are less limited, such as in southerly areas or in response to global warming, populations can quickly adapt to the new conditions by shifting to shorter and/or less intense cycles. Such cycles tend to reflect seasonal variations in breeding with limiting factors including a greater prevalence of generalist predators. The cycles may become of such low amplitude they seem to disappear, though the genetic potential remains.

Population cycling offers survival advantages in harsh conditions, such as preventing overpopulation and resource exhaustion, especially in species capable of rapid breeding. This cycle includes a dispersal phase (Andreassen et al. 2013; Gaines and McClenaghan Jr. 1980; Krebs 1992), aiding in migration to new territories. In some cases, these cycles may help avoid peaks in specialised predators and reduce disease spread.

**FIGURE 3:**
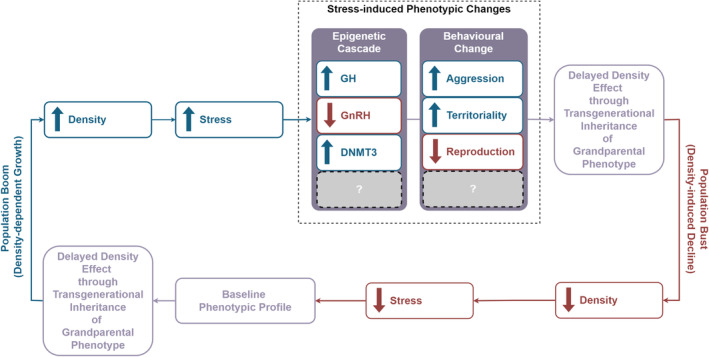
Our proposed epigenetic‐based model for arctic population boom and bust cycles. The figure illustrates the cyclic relationship between population density, stress and phenotypic changes, driven by epigenetic and behavioural modifications, resulting in population booms and busts. Increased population density triggers stress (e.g., food restriction), leading to a cascade of epigenetic changes (including alterations in growth hormone [GH], gonadotropin‐releasing hormone [GnRH], and DNA methyltransferase 3 [DNMT3]) and behavioural changes (increased aggression and territoriality, decreased reproduction). These phenotypic changes can be inherited transgenerationally, which provides an explanation for observed delayed‐density dependence/effects seen before the bust (i.e., it takes several generations for the phenotypic changes leading to the bust to fully manifest). A population boom occurs through density‐dependent growth, the timing of which can be impacted by environmental factors such as predators, while a population bust follows due to the impact of density‐induced stress, completing the cycle (see main text for further details).

Our hypothetical intrinsic program (Figure 3) is mediated through epigenetic changes that target genes associated with sexual development/growth (hormones) and behaviour (see Figure 3 for a detailed proposal and Table 1 for testable hypotheses). Indeed, other biological rhythms including circatidal (Hafker et al. 2023), circadian (Fagiani et al. 2022), circalunar (Neumann and Kaiser 2023) and circannual (Lincoln 2019) all have well‐defined intrinsic mechanisms that drive the cycle. By analogy, multiannual epigenetic rhythms driven by harsh conditions likely contribute to multiannual population cycles.

**TABLE 1:** Testable hypotheses for intrinsic population cycle for lemmings and voles.

Increased levels of growth hormone (GH) produce the Chitty effect	Measure levels of GH as population increases
Stress increases as population increases and resources become limited	Stress hormones have been tested in enclosed populations but not free animals. Test stress hormones as population increases in wild animals
The innate population cycle is largely driven by epigenetic changes	Genome‐wide methylation (epigenetic) studies across the entire population cycle to identify key pathways that explain the changes in animal behaviour. We have commenced this work

Our proposed population cycle begins following the population crash and dispersal of the animals, which occurs following peak population density. At this point, the animals are at their lowest density and lowest levels of stress (Figure 3, lower section). As population numbers increase so does the stress in individual animals. For example, higher population density leads to less food availability, a stressor in itself, therefore individual animals must forage at greater distances increasing their chances of being predated, an additional stressor. Both stress (Dee, Ryznar, and Dee 2023) and calorie restriction (Hernandez‐Saavedra et al. 2019) cause epigenetic changes in various species, including laboratory rodents. Therefore, we propose that both stress and calorie restriction alter the epigenetics of individual animals.

Evidence implicating epigenetic involvement in lemming/vole studies includes increasing levels of growth hormone (GH) as the population increases as part of the “Chitty effect”, a well‐described phenomenon whereby the weight of cycling animals is increased by 20%–30% at the time the population peaks (Boonstra and Krebs 1979; Chitty 1952). Additionally, at high density, there is a reduction in the expression of GnRH in the hypothalamus of offspring (Edwards et al. 2021). The changes in GnRH levels are mediated by epigenetic regulation of the GnRH gene (Edwards et al. 2021) and lead to lower levels of reproduction.

DNMT3 are a family of methylation enzymes involved in regulating many cellular functions (Chedin 2011). Higher levels of GH increase the activity of DNMT3 enzymes in cells (Shafiei et al. 2008). Lower levels of GnRH have also been shown to increase the activity of DNMT3 family members (Coyle et al. 2020; Zhang et al. 2017). Thus, both the increase in GH and decrease in GnRH increase DNMT3 family activity leading to increased methylation (an epigenetic marker that controls gene expression) across the genome.

In our model, environmental factors such as increased predation can influence the time to population peak (density‐dependent growth), consistent with many years of field observation (Figure 3, left‐hand side). Once the population approaches peak (Figure 3, top section), there is increased stress and limited resources. This dramatic change in environment, alongside high levels of DNMT3, triggers an epigenetic cascade (Figure 3, top section). Epigenetic cascade is an emerging concept where a single genetic or environmental event can trigger a cascade of epigenetic changes (Liu et al. 2023). Some of these epigenetic changes are selectively passed down to offspring via established mechanisms (Skinner 2016; Takahashi et al. 2023; Tuscher and Day 2019). These epigenetically regulated genes can fundamentally alter the biology and behaviour of offspring (see Nilsson, Sadler‐Riggleman, and Skinner 2018). We propose that the inheritance of these genes leads to the intrinsic motivational changes and observed behavioural differences associated with population crash and dispersal (Figure 3, top section). Specifically, the selective transgenerational inheritance of increased confrontational motivations manifested as increased aggression and territoriality behaviours, is associated with the dispersal of the population. These epigenetically driven phenotypic changes in offspring eventually accumulate producing a phase change from rising population density to a plateau and eventual collapse.

The timing of these epigenetic changes will require direct research, but they would take several generations (Nilsson, Sadler‐Riggleman, and Skinner 2018). However, there is no consensus around the length of a lemming or vole generation length, with estimates from 1 to 3 generations per year (Ehrich and Jorde 2005) and almost certainly vary during different phases of the cycle. Delayed‐density dependence occurs when populations increase above normal capacity because there is a lag in negative feedback mechanisms, an observation reported for lemmings and voles (Edwards, Boonstra, and Oli 2023). Our model provides an explanation for delayed‐density dependence/effects (Figure 3, top section, right‐hand side) as the inherited epigenetic changes take multiple generations to manifest in the behavioural changes associated with dispersal and a population crash.

**FIGURE A1:**
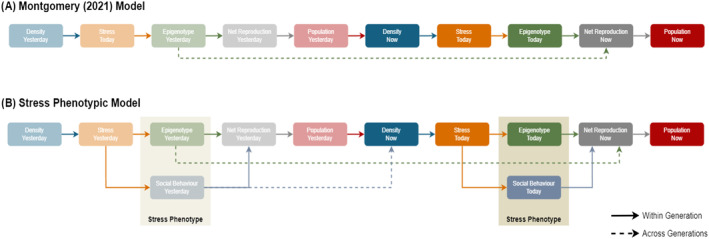
Simplified comparison of the Montgomery (2021) model and the stress phenotypic model for population dynamics across generations. In a simplified form, Montgomery's putative model asserts that (A) stress impacts epigenotype, reducing current and future net reproduction. Our model (B) agrees with these effects but introduces social behaviour as part of a critical extension to an overall stress‐induced phenotype. This phenotype impacts net reproduction through inherited methylation changes and density through aggression and territoriality changes. These separate effects form parallel channels through which the current stress state is transmitted.

This model is a new way of thinking about things (see Figure A1, which highlights the novelty of our model). Extrinsic factors are a critical component of population cycles, but it is the epigenetic changes driven by these extrinsic factors that is the causative agent in driving the cycle. Without epigenetic changes, animals would find a point of equilibrium or display environmental‐induced random population fluctuations rather than having regular cycles.

## 3 Conclusions

One hundred years of research has provided significant insights into Arctic population cycles. However, no universal mechanism that explains the extraordinary regularity of population cycles has been identified. Our proposed intrinsic hypothetical model integrates the complexities of environmental pressures, genetic predispositions and epigenetic modifications. Our model diverges from previous theories by suggesting that epigenetic changes triggered by rising population densities are key intrinsic drivers of arctic population cycles. Previous models primarily attribute these cycles to extrinsic factors and suggest that the few epigenetic changes analysed are a consequence of these extrinsic influences, not a driver. The details of our model will need refining as further data are collected. Integrating epigenetic research with traditional ecological studies promises to deepen our understanding of population regulation, offering insights crucial for conservation and management strategies in a rapidly changing climate. Longer term, understanding the epigenetic changes associated with the behavioural changes in lemmings and voles may provide novel insights into human behaviour.

## Author Contributions

**Elizabeth A. Levay:** visualization (supporting), writing – original draft (supporting), writing – review and editing (equal). **Helen Nasser:** visualization (lead), writing – review and editing (equal). **Matthew D. Zelko:** visualization (supporting), writing – review and editing (equal). **Jim Penman:** writing – review and editing (equal). **Terrance G. Johns:** conceptualization (lead), funding acquisition (lead), visualization (supporting), writing – original draft (lead), writing – review and editing (equal).

## Conflicts of Interest

EAL, HN, MDZ and TGJ are employed by Epigenes Australia, while JP is the Director of Epigenes Australia.

## Data Availability

No data were generated for this paper.
